# EffiCiency and Safety of an eLectronic cigAreTte (ECLAT) as Tobacco Cigarettes Substitute: A Prospective 12-Month Randomized Control Design Study

**DOI:** 10.1371/journal.pone.0066317

**Published:** 2013-06-24

**Authors:** Pasquale Caponnetto, Davide Campagna, Fabio Cibella, Jaymin B. Morjaria, Massimo Caruso, Cristina Russo, Riccardo Polosa

**Affiliations:** 1 Centro per la Prevenzione e Cura del Tabagismo, Azienda Ospedaliero-Universitaria “Policlinico-V. Emanuele”, Università di Catania, Catania, Italy; 2 Institute of Internal Medicine, S. Marta Hospital, Azienda Ospedaliero-Universitaria “Policlinico-V. Emanuele”, Università di Catania, Catania, Italy; 3 National Research Council of Italy, Institute of Biomedicine and Molecular Immunology, Palermo, Italy; 4 Division of Cardiovascular and Respiratory Studies, Hull York Medical School, University of Hull, Castle Hill Hospital, Cottingham, United Kingdom; Centre for Addiction and Mental Health, Canada

## Abstract

**Background:**

Electronic cigarettes (e-cigarettes) are becoming increasingly popular with smokers worldwide. Users report buying them to help quit smoking, to reduce cigarette consumption, to relieve tobacco withdrawal symptoms, and to continue having a ‘smoking’ experience, but with reduced health risks. Research on e-cigarettes is urgently needed in order to ensure that the decisions of regulators, healthcare providers and consumers are based on science.

*Methods* ECLAT is a prospective 12-month randomized, controlled trial that evaluates smoking reduction/abstinence in 300 smokers not intending to quit experimenting two different nicotine strengths of a popular e-cigarette model (‘Categoria’; Arbi Group Srl, Italy) compared to its non-nicotine choice. GroupA (n = 100) received 7.2 mg nicotine cartridges for 12 weeks; GroupB (n = 100), a 6-week 7.2 mg nicotine cartridges followed by a further 6-week 5.4 mg nicotine cartridges; GroupC (n = 100) received no-nicotine cartridges for 12 weeks. The study consisted of nine visits during which cig/day use and exhaled carbon monoxide (eCO) levels were measured. Smoking reduction and abstinence rates were calculated. Adverse events and product preferences were also reviewed.

**Results:**

Declines in cig/day use and eCO levels were observed at each study visits in all three study groups (p<0.001 vs baseline), with no consistent differences among study groups. Smoking reduction was documented in 22.3% and 10.3% at week-12 and week-52 respectively. Complete abstinence from tobacco smoking was documented in 10.7% and 8.7% at week-12 and week-52 respectively. A substantial decrease in adverse events from baseline was observed and withdrawal symptoms were infrequently reported during the study. Participants’ perception and acceptance of the product under investigation was satisfactory.

**Conclusion:**

In smokers not intending to quit, the use of e-cigarettes, with or without nicotine, decreased cigarette consumption and elicited enduring tobacco abstinence without causing significant side effects.

**Trial Registration:**

ClinicalTrials.gov NCT01164072 NCT01164072

## Introduction

Cigarette smoking is the single most important cause of avoidable premature mortality in the world and quitting is known to rapidly reduce risk of serious diseases such as lung cancer, cardiovascular disease, strokes, chronic lung disease and other cancers [1], [2]. The World Health Organization (WHO) Framework Convention on Tobacco Control (FCTC) advises that the key to reducing the health burden of tobacco is to encourage abstinence among smokers [3]. Currently available smoking-cessation medications (including nicotine replacement therapy - NRT, buproprion and varenicline) are known to increase the likelihood of quitting smoking, particularly if combined with counseling programs [4]. However, they lack high levels of efficacy in real-life settings [reviewed in 5]. Consequently, more effective approaches are needed to reduce the burden of cigarette smoking.

E-cigarettes are battery-operated devices designed to vaporize a liquid solution of propylene glycol and/or vegetable glycerin in which nicotine or other aromas may be dissolved [6]. Puffing activates a battery-operated heating element in the atomizer and the liquid in the cartridge is vaporized as a plume of mist that is inhaled. As e-cigarettes do not burn tobacco, these products may be considered a lower risk substitute for factory-made cigarettes [7]. Most e-cigarettes are designed to look like traditional cigarettes and simulate the visual, sensory, and behavioural aspects of smoking traditional cigarettes [7]. Moreover a recent internet survey on the satisfaction of e-cigarette use has reported that the device helped in smoking abstinence and improved smoking-related symptoms [8]. These factors indicate that the e-cigarettes may be an effective and safe cigarette substitute, and therefore merits further evaluation for this purpose.

In two recent case series, we reported objective measures of long-term smoking abstinence in inveterate smokers with severe nicotine dependence and/or major depression who quit after taking up an e-cigarette [9], [10]. Moreover, in a prospective 6-month proof-of-concept study, e-cigarettes were shown to substantially decrease cigarette consumption without causing significant side effects in 40 smokers not intending to quit [11].

Obviously, these products need to be adequately regulated. Thus far, there have been heterogeneous regulatory responses ranging from no regulation to complete bans. In Italy, ‘Categoria’ e-cigarettes (‘Categoria’; Arbi Group Srl, Italy) have been approved for marketing in 2010 by the Italian Institutes of Health (ISS – Istituto Superiore di Sanità). However, the WHO’s Study Group on Tobacco Product Regulation advised a negative approach to e-cigarettes [12]. The basis for this regulatory conclusion is uncertain, and more research on e-cigarettes must be conducted in order to ensure that the decisions of regulators, healthcare providers and consumers are based on science [13]. Consequently, formal appraisal of regular e-cigarette use in relation to reducing tobacco smoking consumption and the possibility of adverse events is now required to confirm and expand preliminary positive findings [9]–[11], [14].

With this in mind, we designed ECLAT, the first randomized controlled trial investigating the EffiCacy and safety of an eLectronic cigAreTte. ECLAT is a prospective 12-month double-blind, controlled, randomized clinical study to evaluate smoking reduction, smoking abstinence and adverse events in smokers not intending to quit experimenting two different nicotine strengths of a very popular e-cigarette brand (‘Categoria’; Arbi Group Srl, Italy). As it was unrealistic to also have a control group specifically for e-cigarette use given the “naturalistic” setting and study population, ECLAT was ‘controlled’ only in relation to the comparison among different nicotine strengths. We also monitored adverse events and measured participants’ perception and satisfaction of the product.

## Methods

### Participants

**Figure 1:**
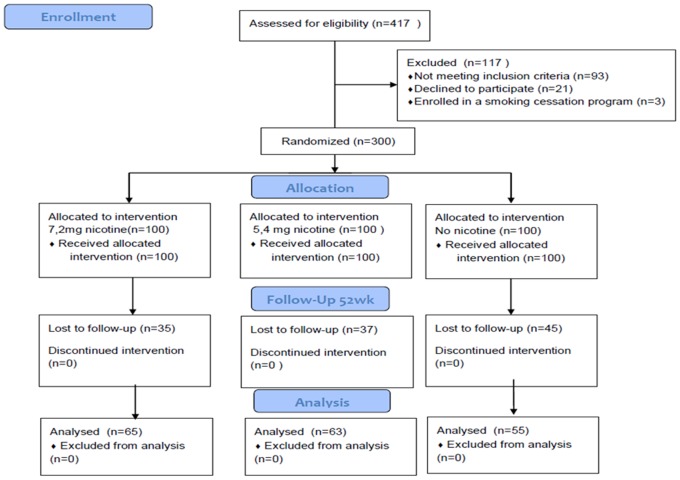
Flow of participants. After screening for the study inclusion/exclusion criteria, a total of 300 regular smokers consented to participate and were included in the study**.** Participants were randomized into three separate study groups (A, B, and C). Participants randomized in **s**tudy group A received 12 weeks supply of “Original” 7.2 mg nicotine cartridges; those in study group B, two 6-week supplies of cartridges, one of the “Original” 7.2 mg nicotine cartridges and a further 6 weeks with supply of “Categoria” 5.4 mg nicotine cartridges; participants in study group C received 12 weeks supply of no-nicotine cartridges (i.e. control).

Regular smokers from Catania (Italy) not intending to quit were recruited during the period June 2010– February 2011 following placement of advertisements in a local newspaper inviting them to try the e-cigarette ‘Categoria’ (Arbi Group Srl, Italy) to reduce the risk of tobacco smoking.The trial profile is presented in Figure 1. It was mentioned that the product was an healthier alternative to tobacco smoke and that could be freely used as a tobacco cigarette substitute, as much as they liked. No other specific instructions were given. Participants were told that they would be randomized to three similar products, but characterized by different nicotine strengths in the cartridge. They were also told that the purpose of the study was to evaluate the chance of reducing tobacco smoking consumption with e-cigarette use, to monitor the possibility of adverse events during the study, and to score their perception and satisfaction of the product. No financial incentive was offered for participation. The first consecutive 300 eligible smokers were included in the study (Centro per la Prevenzione e Cura del Tabagismo - CPCT; Università di Catania, Italy).

Inclusion criteria were: (a) smoke ≥10 factory made cigarettes per day (cig/day), for at least the past five years, (b) age 18–70 years, (c) in good general health; (d) not currently attempting to quit smoking or wishing to do so in the next 30 days (this was verified at screening by the answer “NO” to both questions “Do you intend to quit in the next 30 days?” and “Are you interested in taking part in one of our smoking cessation programs?”), and (e) committed to follow the trial procedures.

Exclusion criteria were: (a) symptomatic cardiovascular disease; (b) symptomatic respiratory disease; (c) regular psychotropic medication use; (d) current or past history of alcohol abuse; (e) use of smokeless tobacco or nicotine replacement therapy, and (f) pregnancy or breastfeeding.

The study was approved by the “Policlinico-Vittorio Emanuele” ethics committee and participants gave written informed consent prior to participation in the study. No deviations were introduced in the protocol approved from ethic committee.

### Products Tested:“Categoria” e-cigarette

**Figure 2:**
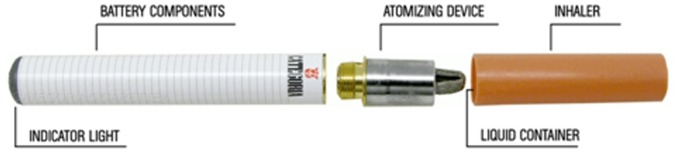
Image of the product tested in the study. The “Categoria” electronic cigarette is a three-piece model consisting of a disposable inhaler/mouthpiece (the cartridge), an atomizer and a rechargeable battery (the cigarette body). Disposable cartridges used in this study looked like tobacco cigarette’s filters containing an absorbent material saturated with a liquid solution of propylene glycol and vegetable glycerin in which different concentrations of nicotine or an aroma were dissolved. The cigarette body contains a rechargeable 3.7 V-90 mAh lithium-ion battery that activates the heating element in the atomizer.

The “Categoria” e-cigarette (model “401”) was used in this study. It is a three-piece model that closely resembles a tobacco cigarette **(** **Figure 2** **).** Its heating element in the atomizer is activated by a rechargeable 3.7 V-90 mAh lithium-ion battery. A fully charged battery can last up to the equivalent of 50–70 puffs.

Disposable cartridges used in this study looked like tobacco cigarette’s filters containing an absorbent material saturated with a liquid solution of propylene glycol and vegetable glycerin in which nicotine or an aroma was dissolved. Disposable cartridges had to fit securely onto the heating element of the atomizer in order to produce a consistent vapour. Three different types of cartridges were provided for the study; “Original” 7.2 mg nicotine (2.27±0.13% nicotine), “Categoria” 5.4 mg nicotine (1.71±0.09% nicotine) and “Original” without nicotine (“sweet tobacco” aroma). Detailed toxicology and nicotine content analyses of these cartridges had been carried in a laboratory certified by the Italian Institute of Health and can be found at: http://www.liaf-onlus.org/public/allegati/categoria1b.pdf.

The “Categoria” electronic cigarette kit and cartridges were provided free of charge by the local distributor, Arbi Group Srl, Italy.

### Study Design

**Figure 3:**
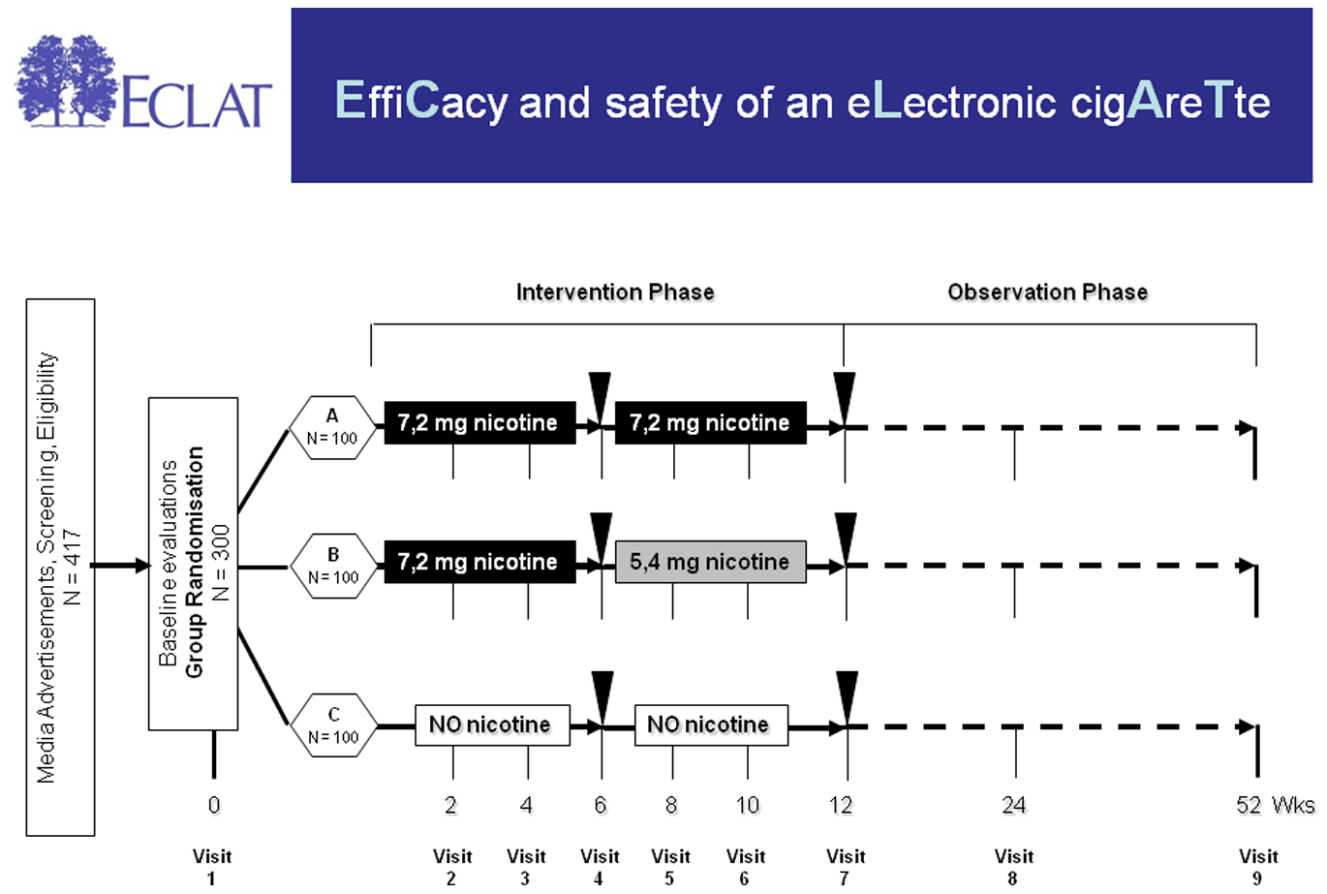
Schematic diagram of the ECLAT study design. Smokers not currently attempting to quit smoking or wishing to do so in the next 30 days were randomized in three study groups**:** group A (receiving 12 weeks of 7.2 mg nicotine cartridges), group B (receiving 6-weeks of 7.2 mg nicotine cartridges and a further 6 weeks with 5.4 mg nicotine cartridges), and group C (receiving 12 weeks of no-nicotine cartridges). Participants in each group were prospectively reviewed for up to 52-weeks during which smoking habits, eCO levels, adverse events, vital signs, and product preference were assessed at each study visits. Additionally, saliva samples were collected at week-6 and at week-12 (closed triangles) for cotinine measurement in those who stated they had not smoked and with an eCO ≤7 ppm.

The study is a three-arms double-blind, controlled, randomized, clinical trial designed to assess the efficacy and safety of ‘Categoria’ e-cigarette loaded with 7.2 mg nicotine and 5.4 mg nicotine cartridges in comparison to no-nicotine cartridges (Figure 3). At baseline, participants were randomized into three separate study groups. The randomization sequence was computer generated by using blocks size of 15 with an allocation ratio of 5∶5:5 for each of the three study conditions (A, B, and C). Participants randomized in study group A received 12 weeks supply of 7.2 mg nicotine cartridges; those in study group B, two 6-week supplies of cartridges, one of the 7.2 mg nicotine cartridges and a further 6 weeks with supply of 5.4 mg nicotine cartridges; participants in study group C received 12 weeks supply of no-nicotine cartridges (i.e. control). Blinding was ensured by the identical external appearance of the cartridges. The hospital pharmacy was in charge of randomization and packaging of the cartridges. A prospective evaluation of efficacy and safety was repeated at two additional follow up visits at 24- and 52-weeks. Thus the study consisted of a total of nine visits: a baseline visit and eight follow up visits (at week-2, week-4, week-6, week-8, week-10, week-12, week-24 and week-52).

### Study Schedule

Participants attended their study visits at the smoking cessation clinic at approximately the same time of day. With the exception of the baseline study day, most visits took approximately 10–15 minutes.

At baseline (study visit 1), socio-demographic factors, and a detailed smoking history were annotated and individual pack-years (pack/yrs) calculated, together with the subjective ratings of depression and anxiety assessed by Beck Depression Inventory (BDI) [15] and Beck Anxiety Inventory (BAI) [16], respectively. Physical dependence and behavioural dependence were measured by Fagerstrom Test for Nicotine Dependence (FTND) [17] and Glover-Nilsson Smoking Behavioral Questionnaire (GN-SBQ) [18], respectively. Additionally, levels of carbon monoxide in exhaled breath (eCO) were measured using a portable device (Micro CO, Micro Medical Ltd, UK). Vital signs, body weight, and adverse events were also recorded at baseline.

Participants were then given a free e-cigarette kit containing two rechargeable batteries, a charger, and two atomizers and instructed on how to charge, activate and correctly use the e-cigarette. Key troubleshooting support was provided and phone numbers were supplied for both technical and medical assistance. A full 2-weeks supply of either nicotine or no-nicotine cartridges (depending on the study arm allocation) was also provided and participants were trained on how to load them onto the e-cigarette’s atomizer. Participants were permitted to use the study product *ad libitum* throughout the day (up to a maximum of 4 cartridges per day, as recommended by the manufacturer) in the anticipation of reducing the number of cig/day smoked, and requested to fill a 2-weeks’ study diary. Study diary sheets were compiled by participants on a daily basis to record details about their daily usage of tobacco cigarette, cartridge use, withdrawal symptoms and adverse events (AEs). In general, study diary sheets allow recording of several items over a 15-day period in one single page and participants received one new study diary sheet every 15 days. To cover a longer period (e.g. 30 or 60 days) multiple pages of 15 days were used. Participants were asked to complete a check list of symptoms likely to be related to tobacco smoking, withdrawal symptoms (i.e. anxiety, depression, insomnia, irritability, constipation, hunger) and/or e-cigarette.

No emphasis on encouragement, motivation and reward for the smoking cessation effort were provided since this study was intended to monitor smokers (not wishing to quit) using e-cigarettes.

Participants were invited to return to our clinic at week-2 (study visit 2), week-4 (study visit 3), week-6 (study visit 4), week-8 (study visit 5), week-10 (study visit 6), and week-12 (study visit 7), a) to receive further free supply of cartridges together with the study diaries for the residual study periods, b) to record their eCO levels, c) to measure vital signs, and d) to return completed study diaries and unused study products. Additionally, saliva samples were collected at week-6 (study visit 4) and at week-12 (study visit 7) for cotinine measurement in those who stated they had not smoked (not even a puff) and with an eCO ≤7 ppm. Participants were asked to chew a small cotton roll (TR0N00RU2, Dentalica, Milano, Italy) for 60 seconds. Cotton rolls were placed into polypropylene tubes and stored at −20°C until use. Saliva samples were analysed in duplicate for cotinine analysis by gas chromatography [19]. At the end of study visit 7, participants were informed that no more cartridges would be provided by the investigators, but that they were advised to continue using their e-cigarette if they wish to do so.

Study participants attended two additional follow up visits at week-24 (study visit 8), and at week-52 (study visit 9) to report product use (cartridges/day) and the number of any tobacco cigarettes smoked (from which reduction and quit rates could be calculated), and to re-check eCO levels.

Adverse events, resting blood pressure, heart rate, and body weight were recorded again as well as participants’ liking of the product (for those participants who were still continuing to use their e-cigarette at week 24 and 52).

During the study we also assessed spirometric data, fractional exhaled NO (FeNO) levels, craving scores, and withdrawal ratings by Minnesota Nicotine Withdrawal Scale-MNWS [20]; these results will be reported in different papers.

### Study Outcome Measures

A ≥50% reduction in the number of cig/day since baseline, defined as self-reported reduction in the number of cig/day compared to baseline [21], was calculated at each study visit (“reducers”).

Abstinence from smoking, defined as complete self-reported abstinence from tobacco smoking - not even a puff (together with an eCO concentration of ≤7 ppm) fsince the previous study visit, was calculated at each study visit (“quitters”). Failing to meet the above criteria defines smoking reduction/cessation failure.

Adverse events, symptoms thought to be related to tobacco smoking and e-cigarette use and to withdrawal from nicotine were annotated at baseline and at each subsequent study visit on the adverse event page of the study diary. Vital signs were also recorded.

Participants’ perception and liking of the product were assessed by asking to rate their level of satisfaction with the products compared to their own cigarettes using a visual analogue scale (VAS) from 0 to 10 points (0 = being ‘completely unsatisfied’, 10 being = ‘fully satisfied’); using the same scale, they also rated how much they missed their own brand (0 = being ‘did not miss it at all’, 10 being = ’missed too much’) and whether they would recommend it to a friend/relative (0 = being ‘not recommended at all’, 10 being = ‘absolutely recommended’) [11].

### Statistical Analyses

This was a proof-of-concept pilot study, the first of its kind, hence no previous data was available for power calculation. In our preliminary work with “Categoria” e-cigarettes supplied with 7.4 mg nicotine cartridges, we reported a quit rate of 22.5% at 6-months in smokers not wishing to quit and with an observed attrition rate of 32.5% (11). Assuming a 10% difference in success rate between the two nicotine arms (A and B) and the arm without any nicotine addiction (C), we estimated that a sample of 93 subjects for each arm would have been adequate for the study, with a type I error of 0.05 and type II error of 0.25. Consequently, we recruited 100 subjects for each arm of the study.

Study outcome measures were computed by including all enrolled participants - assuming that all those individuals who were lost to follow-up are classified as failures (intention-to-treat analysis). In per-protocol analyses enrolled subjects who did not drop-out were evaluated.

Normality of variable distributions was evaluated by Kolmogorov-Smirnov Test. Parametric and non-parametric data were expressed as mean (±SD) and median (and interquartile range [IQR]) respectively. Within-group (from baseline) and between-group differences were evaluated by means of parametric and non parametric statistical tests, for paired and unpaired data, as appropriate. Significance of differences in frequency distribution of categorical variables were tested by χ^2^ test. Correlations between variables were calculated using Spearman’s rank correlation. Statistical methods were 2-tailed, and P values of <0.05 were considered significant.

The analyses were carried out using Statistical Package for Social Sciences (SPSS Inc., Chicago, IL) for Windows version 17.0.

## Results

### Participant Characteristics

After screening for the study inclusion/exclusion criteria, a total of 300 [M 190; F 110; mean (±SD) age of 44.0 (±12.5) years] regular smokers (median [IQ range] pack/yrs of 24.9 [14.0–37.0]) consented to participate and were included in the study (**Table 1**).

**Table 1:** General characteristics of ECLAT study sample at baseline.

	Overall sample (N = 300)	Group A (N = 100)	Group B (N = 100)	Group C (N = 100)	P
Males/Females (No)	190/110	61/39	66/34	63/37	NS
Age (yrs ± SD)	44.0±12.5	45.9±12.8	43.9±12.2	42.2±12.5	*
Age at initiation (mean ± SD)	16.8±3.9	16.4±3.9	17.3±4.3	16.9±3.5	NS
Education level (No. [%]):					
Low	93 (31%)	28 (28%)	32 (32%)	33 (33%)	
Intermediate	160 (53%)	57 (57%)	59 (59%)	44 (44%)	0.055
High	47 (16%)	15 (15%)	9 (9%)	23 (23%)	
Pack/yr (median [IQ range])	24.9 (14.0–37.0)	24.0 (14.3–37.0)	25.3 (16.9–38.8)	25.5 (12.0–35.0)	NS
Cig/day (median [IQ range])	20.0 (15.0–25.0)	19.0 (14.0–25.0)	21.0 (15.0–26.0)	22.0 (15.0–27.0)	NS
Past attempts to quit (% yes)	51	56	48	47	NS
Number past attempts to quit (mean ± SD)	0.6±0.7	0.7±0.8	0.5±0.6	0.6±0.7	NS
eCO (median [IQ range])	20.0 (15.0–28.0)	19.0 (15.5–29.0)	22.0 (16.0–29.0)	19.5 (14.0–28.0)	NS
FTND (mean ± SD)	5.8±2.2	5.6±2.3	6.0±2.1	5.8±2.2	NS
GN-SBQ (mean ± SD)	20.0±7.2	20.5±7.0	20.5±7.5	19.0±7.2	NS
BDI (median [IQ range])	6.0 (2.0–12.0)	7.0 (2.0–12.5)	6.0 (3.0–12.5)	5.0 (1.0–11.5)	NS
BAI (median [IQ range])	7.0 (3.0–14.0)	7.0 (3.0–14.5)	8.0 (3.0–14.0)	6.5 (2.0–15.5)	NS

Legend: SD – standard deviation; IQR – interquartile range; Pack/yrs – pack-years; Cig/day – Cigarettes smoked per day; eCO – exhaled carbon monoxide; FTND – Fagerstrom Test of Nicotine Dependence; GN-SBQ- Glover-Nilsson Smoking Behavioral Questionnaire; BDI – Beck Depression Inventory; BAI – Beck Anxiety Inventory.

Data are reported for the overall sample and separately for each treatment group. Differences among groups were evaluated by χ^2^ test for categorical variables, one-way analysis of variance (ANOVA) and Fisher protected LSD for parametric variables, and Kruskal-Wallis test for non parametric variables.

* p = 0.04 between A and C groups (ANOVA).

Baseline characteristics between study groups A, B, and C were not significantly different from each other, with the exception of participants’ age in group A vs group C (45.9±12.8 vs 42.2±12.5; p = 0.04, Fisher’s least significant difference).

Two-hundred-twenty-five subjects (75.0%) returned at week-12, 211 (70.3%) at week-24, and 183 (61.0%) for their final follow-up visit at week-52. Baseline characteristics of those who were lost to follow-up were not significantly different from participants who completed the study, with the exception of gender: at week-52, males were 71% of subjects lost to follow-up, while 58% among those still present at week-52 (p = 0.03, χ^2^ test). No significant difference was evident in drop-out rates among study groups at any Study Visit (χ^2^ test).

### Outcome Measures

**Figure 4:**
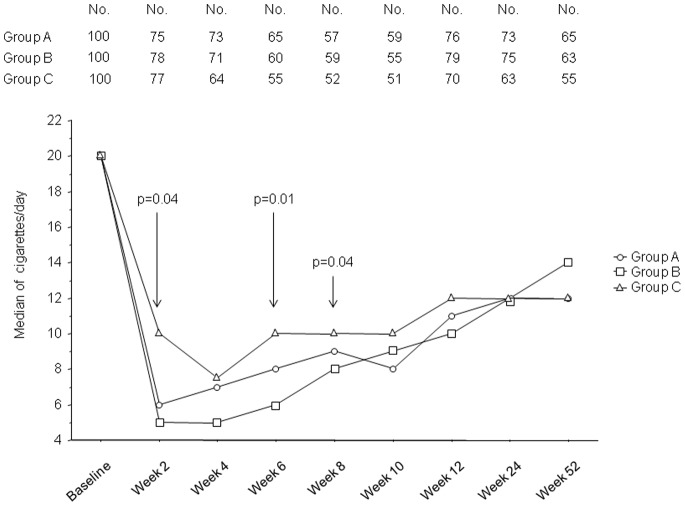
Time-course of changes in the median number of cigarettes/day use from baseline, separately for each study group. A significant reduction (per-protocol evaluation, p<0.0001, Wilcoxon signed-rank test) was observed at each study visits in all three study groups. When significant, between-group differences were indicated (Kruskal-Wallis test). The upper part of the figure illustrates the number of subjects attending each study visit.

A significant reduction of median value (per-protocol evaluation, p<0.0001, Wilcoxon signed-rank test) in cig/day use from baseline was observed at each study visits in all three study groups (**Figure 4**); the median values (and IQR) of cig/day use were 19.0 (14.0–25.0) in Group A, 21.0 (15.0–26.0) in Group B, and 22.0 (15.0–27.0) in Group C at baseline; 11.0 (5.0–15.0), 10.0 (2.3–18.0), and 12.0 (7.0–18.0) respectively at Week-12; 12.0 (5.8–20.0), 14.0 (6.0–20.0), and 12.0 (9.0–20.0) respectively at week-52. Between-group differences in cigarette/day use were significant at week-2, -6, and -8 (Kruskal-Wallis test). When Quitters were excluded from the analysis, at Week-12 median values (and IQR) of cig/day use were 12.0 (7.8–16.0) in Group A, 13.0 (7.0–20.0) in Group B, and 12.0 (8.0–18.0) in Group C (p = 0.65); at Week-52 the same figures were 15.0 (10.0–20.0), 15.0 (10.0–20.0), and 13.0 (10.–20.0) respectively (p = 0.57).

**Figure 5:**
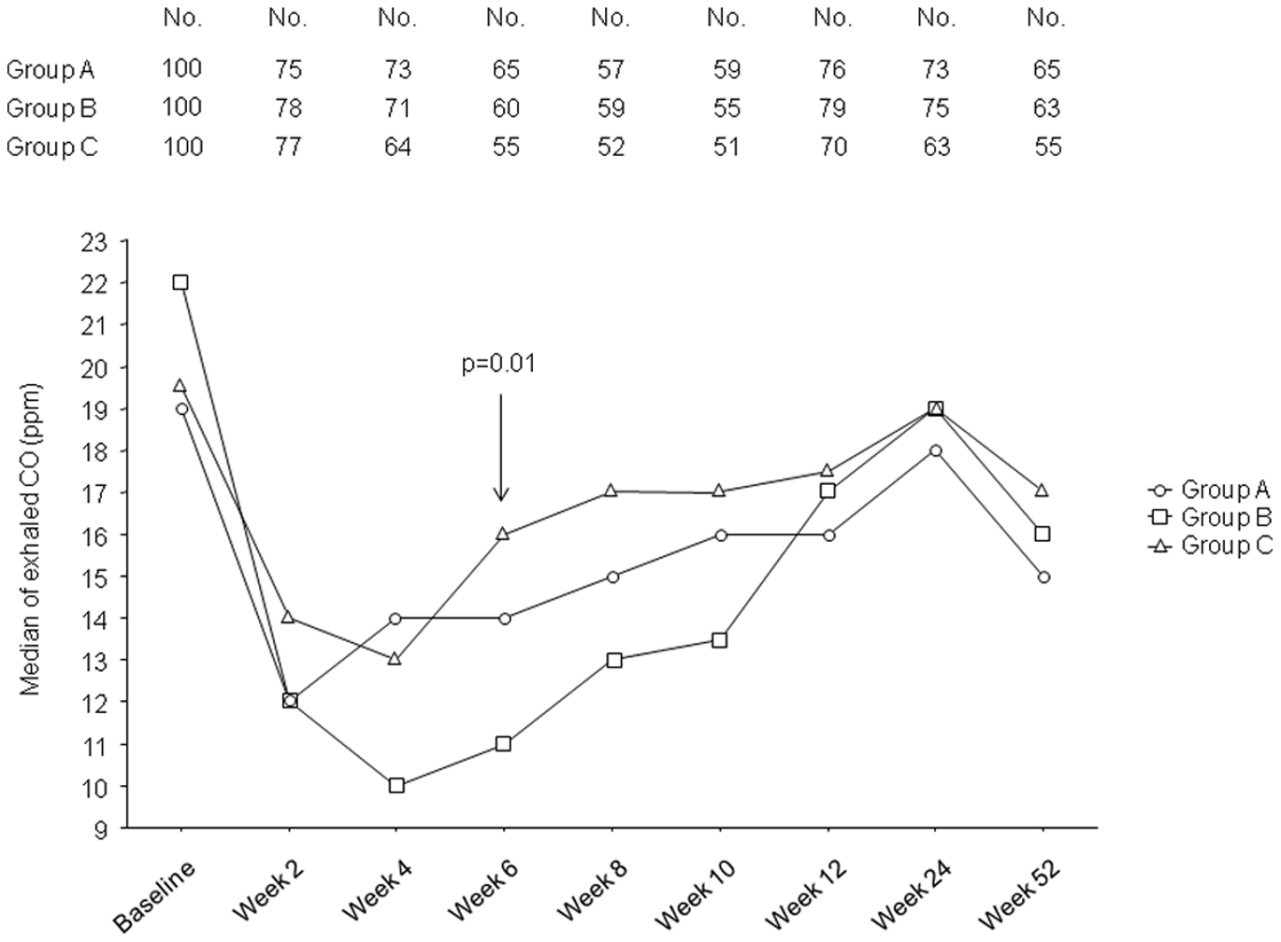
Time-course of changes in the median exhaled CO levels from baseline, separately for each study group. A significant reduction (per-protocol evaluation, p<0.0001, Wilcoxon signed-rank test) was observed at each study visits in all three study groups. When significant, between-group differences were indicated (Kruskal-Wallis test). The upper part of the figure illustrates the number of subjects attending each study visit.

Likewise, a significant reduction (per-protocol evaluation, p<0.0001, Wilcoxon signed-rank test) in eCO levels from baseline was observed at each study visits in all three study groups (**Figure 5**); significant between-group differences were only observed at week-6 (p = 0.01, Kruskal-Wallis test). At Week-12, median (and IQR) eCO values (ppm) were 16.0 (8.0–22.0) in Group A, 17.0 (7.3–24.8) in Group B, and 17.5 (11.0–23.0) in Group C (p = 0.48); at Week-52 they were 15.0 (8.8–29.0), 16.0 (10.0–26.5), and 17.0 (11.3–25.0) respectively (p = 0.93). When excluding Quitters from the analysis, at Week-12 we found 18.0 (9.0–23.0) in Group A, 19.0 (12.0–28.0) in Group B, and 18.0 (13.0–23.0) in Group C (p = 0.48); similarly, at Week-52 they were 18.0 (9.0–23.0), 18.0 (10.–26.0), and 19.0 (13.0–28) respectively (p = 0.59).

Reduction and quit rates (%) during the course of the study are shown separately for each study groups on intention-to-treat analysis in **Table 2**. With a few exceptions, no significant difference was observed among study groups. In particular, at week-12 quitters were 11% in Group A, 17% in Group B, and 4% in Group C. At week-52 the same figures were 13%, 9%, and 4%, respectively. In the subsequent analyses of reduction and quit rates we have combined Groups A and B together for comparison to Group C.

**Table 2:** Reduction and quit rates at different time points, shown separately for each study group (intention-to-treat analysis).

	Reduction rates (%)			Quit rates (%)			
Groups	A	B	C	A	B	C	p value*
Week-2	29.0	38.0	36.0	20.0	12.0	5.0	0.02
Week-4	29.0	33.0	29.0	14.0	14.0	6.0	0.25
Week-6	24.0	26.0	25.0	11.0	15.0	2.0	0.03
Week-8	23.0	21.0	20.0	9.0	12.0	4.0	0.31
Week-10	26.0	15.0	19.0	7.0	15.0	3.0	0.01
Week-12	26.0	20.0	21.0	11.0	17.0	4.0	0.04
Week-24	17.0	19.0	15.0	12.0	10.0	5.0	0.39
Week-52	10.0	9.0	12.0	13.0	9.0	4.0	0.24

* p values are relevant to the differences in frequency distribution in reduction and quit rates among groups at each Study Visits (χ^2^ test).

Excluding quitters, on an intention-to-treat basis a ≥50% reduction in the number of cig/day from baseline was documented in 46/200 (23.0%) subjects in Groups A-B and in 21/100 (21.0%) in Group C at week-12 (p = 0.70, χ^2^ test). At week-52 reducers were 29/200 (14.5%) in Groups A-B and in 12/100 (12.0%) in Group C (p = 0.55). Of these tobacco smoke reducers, 9/200 (4.5%) could be classified as sustained heavy reducers (at least 80% reduction in the number of cig/day) in Groups A-B and 2/100 (2.0%) in Group C at week-12 (p = 0.28); at week-52 sustained heavy reducers were 2/200 (1.0%) in Groups A-Band 2/100 (2.0%) in Group C (p = 0.48).

**Figure 6:**
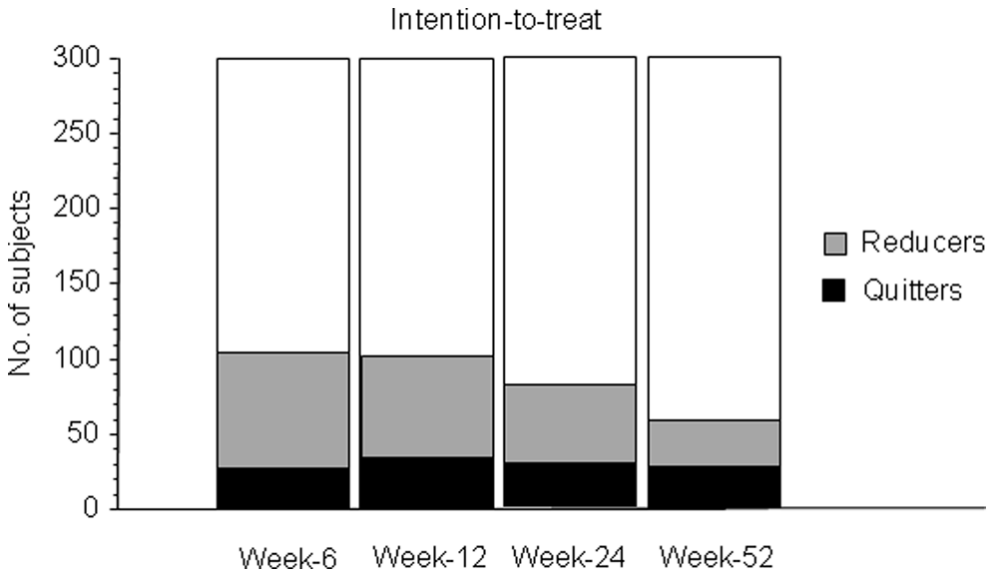
Time-course (at Week-6, -12, -24, and -52) of changes in the number of reducers and quitters in the ECLAT study (intention-to-treat analysis; all three study groups combined together).

On an intention-to-treat basis, at Week-12 complete abstinence from tobacco smoking was documented in 28/200 (14.0%) in Groups A-B and in 4/100 (4.0%) in Group C (p = 0.008, χ^2^ test). At week-52, quitters were 22/200 (11.0%) in Groups A-B and 4/100 (4.0%) in Group C (p = 0.04). (Figure 6). Of these quitters, 7/26 (26.9%) were still using the e-cigarette by the end of the study.

Overall, on an intention-to-treat basis, combined ≥50% smoking reduction and complete abstinence from smoking was shown in 99/300 (33.0%) at week-12 and 57/300 (19.0%) at week-52. The mean (±SE) overall cig/day consumption at baseline was of 21.4 cig/day (±0.5): it decreased to 13.9 cig/day (±0.7) at week-52 (p<0.0001, Wilcoxon signed-rank test).

Switching from 7.2 mg nicotine to 5.4 mg nicotine cartridges at week-6 in study group B did not have any effect; reduction and abstinence rates remained substantially similar in group A and B on all subsequent visits: at week-6, quit rates were 11/100 in Group A and 15/100 in Group B, reduction rates 24/100 and 26/100 respectively; at week-12 quit rates were 11/100 in Group A and 17/100 in Group B, reduction rates 26/100 and 20/100 respectively.

**Figure 7:**
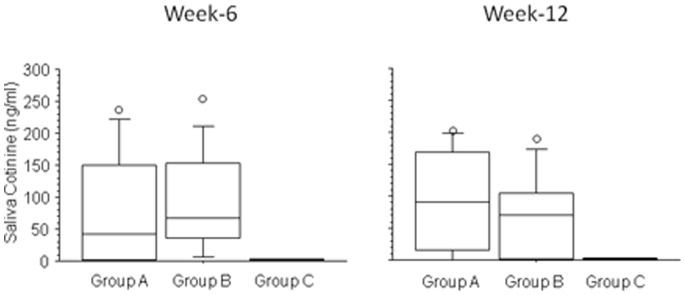
Box plots representation of the changes in saliva cotinine levels measured at week-6 and at week-12 in those who stated they did not smoke and with an eCO≤7 ppm; no significant difference between groups A and B at both time points was found (Mann-Whitney U test). Bars indicate (from the bottom to the top) 10^th^, 25^th^, 50^th^ (median), 75^th^, and 90^th^ percentiles. Values below 10^th^ and above 90^th^ percentiles (outliers) are shown as circles.

Saliva cotinine levels at week-6 and at week-12– in those who stated they did not smoke (not even a puff) and with an eCO ≤7 ppm – were not significantly different between group A and B (**Figure 7**, Mann-Whitney U test); their median (IQR) concentration being 42.5 ng/ml (1.0–149.3) in Group A and 67.8 (35.4–153.0) ng/ml in Group B at week-6, and 91.0 ng/ml (16.3–169.4) in Group A and 69.8 (0.9–104.9) ng/ml in Group B at week-12. As expected, saliva cotinine levels in the no-nicotine group (group C) were barely measurable at week-6 and -12 (**Figure 7**).

### Product Use

Correlations between saliva cotinine levels and number of cartridges/day were highly significant for study groups A and B, at week-6 (Rho = 0.90 for group A, p = 0.005; Rho = 0.74 for group B, p = 0.006, Spearman’s rank correlation) and at week-12 (Rho = 0.93 for group A, p<0.003; Rho = 0.95 for group B, p<0.0004). Details of median (and IQR) cartridge use throughout the study is shown in **Table 3**. At each visit, smoking reduction/cessation failures used significantly less cartridges with respect to reducers and quitters. By and large, no significant difference among groups was observed in terms of cartridge use.

**Table 3:** Details of median (interquartile range - IQR) cartridge use at different time points for the total sample and separately for smoking failures, reducers, and quitters categories.

No. of cartridges	Total sample	Failures	Reducers	Quitters	p value*
Week 2	2 (1–3)	1 (1–3)	2 (1–3)	3 (2–3)	0.0018
Week 4	2 (1–3)	1 (1–3)	2 (1–3)	2 (1–3)	0.0014
Week 6	2 (1–4)	1 (0–3)	3 (1–4)	2 (2–4)	<0.001
Week 8	2 (1–4)	2 (1–3)	3 (2–4)	3 (1–4)	0.0063
Week10	2 (1–4)	2 (0–3)	2 (1–4)	4 (2–4)	0.0002
Week 12	2 (0–3)	1 (0–2)	3 (1–4)	2 (0–4)	<0.0001
Week 24	0 (0–2)	0 (0–1)	1 (0–4)	1 (0–3)	<0.0001
Week 52	0 (0–0)	0 (0–0)	1 (0–3)	0 (0–0)	0.0056

* p values are relevant to the differences in the number of used cartridges among failures, reducers, and quitters subgroups at each time point (Kruskal-Wallis test).

### Safety

**Figure 8:**
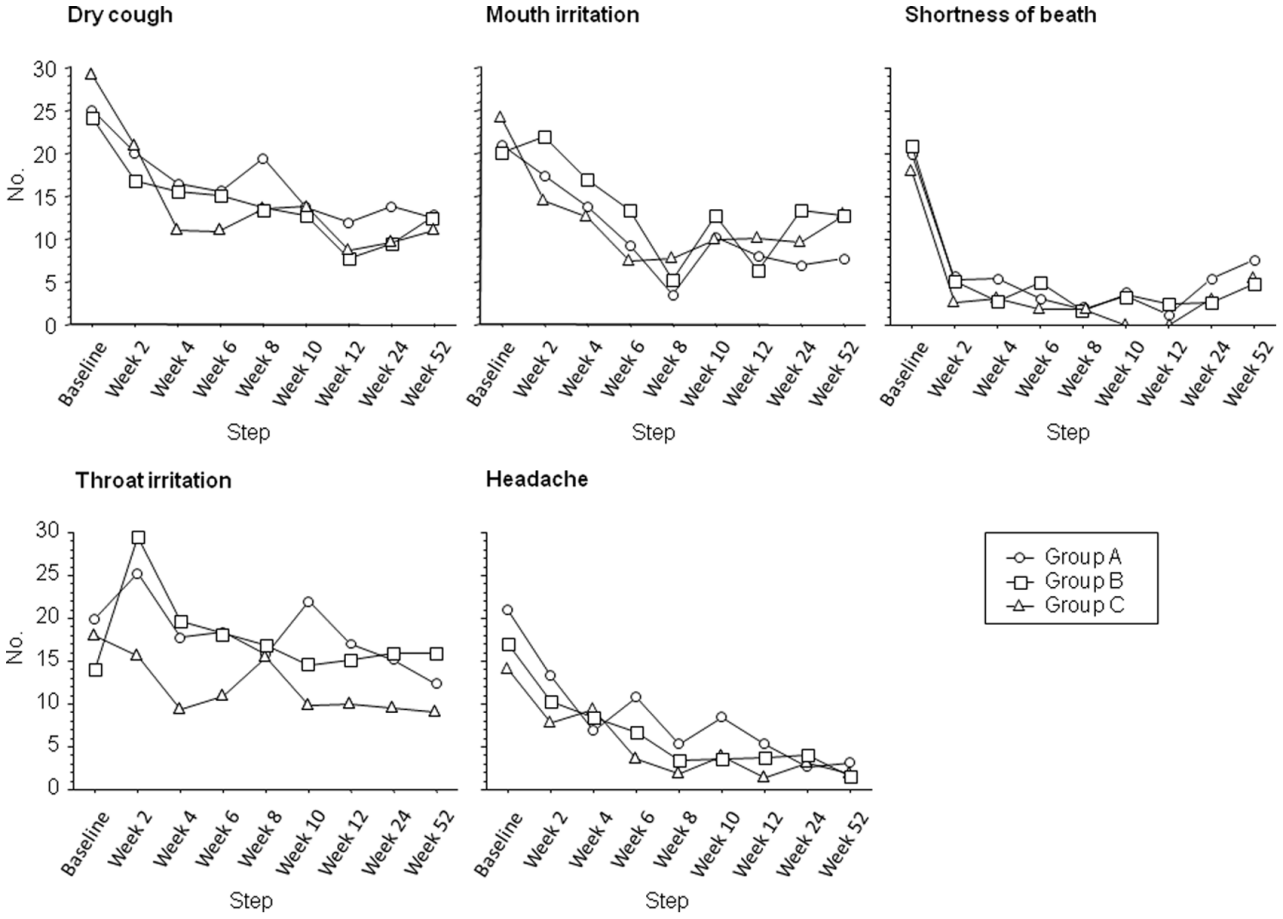
Time-course of changes in the frequency of the five most commonly reported adverse events (AEs) from baseline, separately for each study group. On Y-axis, the number of subjects reporting AEs is depicted. Compared to baseline, a significant reduction in frequency of cough, dry mouth, shortness of breath, and headache was observed at each study visits in all three study groups (per-protocol evaluation, p<0.001, χ^2^ test). No difference was found in frequency distribution of AEs among study groups (χ^2^ test).

Safety analyses included all participants who were using the product at their scheduled visit. **Figure 8** shows the frequency distribution (%) of the five most commonly reported adverse events (AEs), separately for each study groups. Before using e-cigarettes, at baseline, the most frequently reported AEs were cough (26%; average for all study groups combined), dry mouth (22%), shortness of breath (20%), throat irritation (17%), and headache (17%). We performed a between-group evaluation at baseline, at week-12 and at week-52; no difference was found in frequency distribution of AEs among study groups at all the three time-points (χ^2^ test). However, for all the investigated AEs, a significant reduction in frequency of reported symptoms was observed compared to baseline (**Figure 8**). Of all symptoms that progressively decreased throughout the study with the use of e-cigarettes, shortness of breath was substantially reduced from 20 to 4% already by week-2.

Remarkably, side effects commonly recorded during smoking cessation trials with drugs for nicotine dependence were infrequently reported in the course of the study; for example at week-2 hunger, insomnia, irritability, anxiety, and depression were reported by 6.5%, 4%, 3.5%, 3% and 2% participants respectively. Moreover, no serious adverse events (i.e. major depression, abnormal behaviour or any event requiring unscheduled visit to the family practitioner or hospitalisation) occurred during the study.

No significant changes in mean (±SE) body weight, resting heart rate, and systolic/diastolic blood pressure from baseline to the end of the study were observed. Likewise, no significant difference was found among the three study groups throughout the study.

### Product Preferences

The satisfaction level for the product under investigation was not particularly high, the median (IQR) VAS values being 4 (2–5), 4 (1–5) and 3 (1–5) at week-12, -24, and -52 respectively, with no significant difference among the three study groups.

Using the same scale, when participants rated how much they missed their own brand, median (IQR) VAS values were 6 (4–8), 6 (4–8) and 6 (4–8) at week-12, -24, and -52 respectively. No significant difference was found among the three study groups.

Participants were inclined to recommend the e-cigarette to friends or relatives, the median (IQR) VAS values being 7 (5–9), 6 (4–9) and 7 (4–8) at week-12, -24, and -52 respectively. Once again, no significant difference was observed among the three study groups.

## Discussion

The e-cigarette is a very controversial topic, which calls for a balanced analysis of the risks and benefits of these products. Currently, only limited evidence is available and rigorous research on e-Cigarettes is required to guide the decisions of regulators, healthcare providers and consumers. Here, we present the results of ECLAT, the first randomized controlled trial addressing the impact of e-Cigarette use in relation to smoking reduction, smoking abstinence and safety long-term. ECLAT reveals important and persistent modifications in the smoking habits of 300 smokers (not intending to quit) using e-cigarettes, resulting in significant smoking reduction and smoking abstinence. These positive findings were associated with a substantial decrease in adverse events. Moreover, a limited evaluation of withdrawal symptoms indicates that they were reported only occasionally.

Based on our previous experience with smoking cessation media campaigns, the large participation in ECLAT following placement of advertisements in a local newspaper was unexpected. This was driven by an important factor: curiosity. Please note that advertisements were promoted in 2010 when – at least in Italy – the level of awareness of e-cigarettes was very low. Thus, it is more plausible that subjects took interest in the study because they were simply curious about a new electronic product looking like a cigarette and wanted to try it on. For this reason, we are confident that participants enrolled in ECLAT were not interested in quitting.

Soon after inclusion in the study, smokers substantially reduced cig/day use from baseline by more than 50% in all three study groups and this was coupled by reductions in eCO levels. The level of reduction in cig/day use reported here is in agreement with those reported in surveys of e-cigarette users [8], [22], [23] and in our earlier work with the same product [11]. The observed reduction in cig/day use appears to be unrelated to the nicotine content in the cartridges, the non-nicotine study group (C) behaving like both nicotine groups (A and B) at most time-points. This was unpredicted, bringing into question the key function of nicotine in cigarette dependence and suggesting that other factors such as the rituals associated with cigarette handling and manipulation may also play an important role [24], [25].

The percentage decrease in cig/day use from baseline was greater that the percentage decrease in eCO. Besides the obvious element of compensation (i.e. more intense puffing) when smoking fewer cig/day, there is also the possibility that a variability in the time lapse from the last cigarette smoked before eCO measurements may introduce inconsistency (i.e. higher than expected eCO values).

Switching to e-cigarettes resulted in significant smoking reduction and smoking abstinence with a substantial number of quitters (26.9%) still using these products by week-52.

Of note, those who abstained completely from tobacco from the beginning of the study were more likely to stay quit at subsequent follow-ups, whereas those who at first became reducers (dual users) were more likely to relapse later on in the study. Quit rates in the control group (C) were consistently lower at each visit, with a difference that was statistically significant for the most part of the intervention phase of the study. This seems to be in contrast with the earlier interpretation of the observed reduction in cig/day use being unrelated to the nicotine content (discussed earlier). Indeed, saliva cotinine levels in those who had completely switched to the e-cigarette were measurable only in those belonging to groups A and B (and markedly correlates with the number of cartridges/day), however with the exception of a handful of participants, saliva cotinine levels were well below the concentration threshold considered to be representative for regular smokers [26] or experienced e-cigarette users [27]. This is not surprising considering that the model under investigation is not very efficient at delivering nicotine [28]. Furthermore, this product is equipped with a small 90 mAh lithium-ion battery that allows (on a full charge) only about 50–70 puffs. Newer models are now equipped with much higher voltage batteries, thus allowing thicker vapour and up to 500 puffs. Last but not least, technical issues (es. malfunctions) were not uncommon with the model under investigation. In our opinion, it is likely that with this underperforming model all three study groups were similarly behaving as controls, with a minor advantage in quit/reduction rates seen in study group A and B is essentially due to other factors mainly associated to participants’ satisfaction/pleasure such as product’s taste/flavour. In the present investigation, the “sweet tobacco” aroma of the cartridges used in study group C was considered unpleasant by a large number of respondents (18/25; 72%) compared to the other 2 groups (37.8% and 26.7% in group B and A, respectively). To this end, it is interesting to note that smoking reduction/cessation failures used significantly less cartridges with respect to reducers and quitters at each visit.

Given that all smokers were - by inclusion criteria - not interested in quitting, and that the model under investigation was underperforming the rates reported in the present study are impressive. It is possible that for some participants, satisfaction from e-cigarette use was good enough to compensate for their need of own brand cigarette. Indeed the replacement of the ritual of smoking gestures and cigarette handling, the opportunity to use the product in public places and to reduce bad smell, as well as the perception of an improved general sense of wellbeing might have been the cause for the substantial success rates of the ECLAT study.

Although ECLAT findings are not directly comparable with classic cessation and/or reduction studies because of its design (unlike these studies, the ECLAT study sample was characterized by participants selected specifically for their lack of interest in quitting and the subjects were not encouraged to quit smoking, nor provided any help), the observed 52-week abstinence rate appears to be similar to that published in the medical literature with first-line medications for nicotine dependence [29], [30]. However, it cannot be excluded that some of the participants were in fact unintentionally ready to quit given that no formal assessment of their readiness to quit was carried out.

ECLAT is also the first study to address the impact of e-cigarette use in relation to long-term safety. At study outset, typical smokers’ symptoms were documented, but use of “Categoria” e-cigarettes resulted in significant progressive health improvements with no difference among study groups. Specifically, of all symptoms that progressively decreased throughout the study with the use of the product, shortness of breath was substantially reduced (from 20 to 4%) already by week-2.

Although withdrawal symptoms were determined as part of the AEs adverse events assessment, hunger, insomnia, irritability, anxiety, and dysphoric or depressed mood) were uncommon. Withdrawal symptoms are know to be responsible for the impaired ability to achieve and sustain abstinence [31]. It is possible that the e-cigarette by providing a coping mechanism for conditioned smoking cues could mitigate withdrawal symptoms and the desire to smoke associated with smoking reduction and smoking abstinence [32]–[34]. Moreover, e-cigarettes appear to improve cognitive effects during tobacco abstinence [34]. Taken together these mechanisms suggest that e-cigarettes may act as an efficient relapse prevention tool thus providing a plausible explanation for the reduction/cessation rates observed in ECLAT. However, although assessment of symptoms in ECLAT was meticulous, we cannot exclude some degree of recall bias and the reported lack of withdrawal symptoms in the study participants should be considered with caution.

Objective assessment of vital signs were recorded at baseline and at each subsequent study visit. In the ECLAT study, we reported no changes in resting heart rate, and systolic/diastolic blood pressure. Moreover, no serious adverse events (i.e. major depression, abnormal behaviour or any event requiring unscheduled visit to the family practitioner or hospitalisation) occurred during the study.

Notably, no weight gain was observed in the ECLAT sample. This is somewhat surprising given that smoking cessation is typically associated with significant increase in body weight [35]. Thus, the ‘Categoria’ e-cigarette might not only be a safer alternative to smoking tobacco, but can also reduce cigarette consumption with no weight concern.

Carbon monoxide (CO) is a toxic gas and high concentrations are known to be generated during cigarette combustion. Hence, exhaled CO has been universally adopted as a biomarker of exposure to cigarette smoke. Thus, it was not surprising to observe that the smoking reduction/abstinence achieved with use of ‘Categoria’ e-cigarette was associated with a significant decrease in exhaled CO level from baseline. This is in agreement with previous acute studies with a number of different models [32], [33] and in net contrast with other electronic nicotine delivery devices (ENDDs) such as Eclipse (which has been shown to generate substantial levels of eCO) [36].

By the end of the study, 26.9% of quitters were still using e-cigarettes; consequently 73.1% of the quitters were completely freed from their smoking dependence. Thus the large majority of smokers who were successful in quitting using the e-cigarette were successful not only in quitting smoking, but in eventually stopping use of the e-cigarette as well. This is surprising and contradicts the popular conception that the e-cigarette is not effective because people are substituting one addiction for another. In trying to provide an explanation, we noticed that once smokers who were successful in quitting using the e-cigarette realized that they did not need tobacco smoking anymore, they could choose not to smoke and/or use the product. Hence, e-cigarette use played a role by boosting smokers confidence in their ability to quit. However, it must be also noted that, participants who later discontinued the use of e-cigarette went back to smoking their own brand, suggesting that dynamic changes in motivation levels may have occurred in both directions in ECLAT with smokers losing or acquiring confidence in their ability to quit at different time points.

Collectively, the evidence that e-cigarettes helps reducing cigarette consumption and elicits enduring tobacco abstinence without causing significant side effects in individuals unable or not wishing to quit can be seen as an emerging novel approach to tobacco harm reduction [37]. Cigarette smokers, who consider their tobacco use a recreational habit that they wish to maintain in a more benign form, rather than a problem to be medically treated, may have the option of switching to a less harmful source of nicotine. In addition, the current findings of ECLAT and recent research with e-cigarettes [9]–[11] indicates that these products may also be attractive in managing smokers who are not ready to repeat a quit attempt and decline further assistance after relapse [38].

The model under investigation sufficiently well rated on a range of subjective indicators of users’ perception and satisfaction among all study groups. Satisfaction level, in particular, indicates that room for improvement is needed and that the product was not performing adequately as cigarette substitute. Many respondents complained of the frequent failures, lack of durability, difficulty of use (it takes time to familiarize with the puffing technique), and poor taste of the product tested. This is likely to have affected the level of satisfaction with the product and consequently might have been the cause for the number of lost to follow-up and reduction/smoking failures. Nonetheless, participants were prone to recommend the “Categoria” e-cigarette to friends and/or relatives.

When interpreting the outcomes of the ECLAT study, we need to take into considerations some factors. First, because of its unusual design (e.g. smokers not wishing to quit) it is not an ordinary cessation study, hence direct comparison with other smoking cessation products cannot be made.

Second, study design was mainly based on the concept that nicotine is of main importance in dictating smoking addiction, but lacked of a control group specifically for e-cigarette use, We considered unrealistic to have a control for e-cigarette use *per se* in a study in which smokers were not interested in quitting. However, to provide an idea of the size of the effect, please consider that the quit rate of up to 8.7% at 1-year follow up in ECLAT compares very favorably with the national average cessation rate of 0.02% on an yearly basis over the 2001–2011 period in the general population (www.istat.it). We are confident that these findings cannot simply relate to participants self-selection; a genuine effect in term of reduction in tobacco consumption is shown with regular use of these products. Third approximately 40% of the participants failed to attend their final follow-up visit, however this is not unexpected in a smoking cessation study [39]. Fourth, failure to complete the study and several smoking cessation failures could be due to the frequency of technical issues (e.g. e-cigarette malfunctions). Fifth, at time of writing the product tested in ECLAT (model “401”) has become obsolete and is underperforming compared with current models. This model is now discontinued from production. However, when the study was first designed in 2009, it was the only option available to us to investigate an e-cigarette. In future, it will be interesting to compare present findings with those obtained with newer models. Sixth, findings with the product tested in ECLAT cannot be extended to other models and in particular to those belonging to higher quality range. Last but not least, the findings reported from urban Sicilian residents in ECLAT may not be valid for other population samples as it must take into account specific socio-cultural conditions (e.g. the so-called “coffee puff-break” is still considered norm despite the antismoking legislation in Italy).

### Conclusions

The results of this study demonstrate that e-cigarettes hold promise in serving as a means for reducing the number of cigarettes smoked, and can lead to enduring tobacco abstinence as has also been shown with the use of FDA-approved smoking-cessation medications [4], [21]. In view of the fact that subjects in this study had no immediate intention of quitting, the reported overall abstinence rate of 8.7% at 52-week was remarkable. In comparison, in a study of varenicline in smokers that were motivated to quit, the group treated with 1.0 mg twice per day experienced a 52-week quit rate of 14.4% versus 4.9% in the control group [30]. Moreover, these positive results were obtained together with an important reduction in frequency of reported symptoms. Although, these data are promising, they are not definitive and more research about long term safety of these products is still required.
