# Correction: EffiCiency and Safety of an eLectronic cigAreTte (ECLAT) as Tobacco Cigarettes Substitute: A Prospective 12-Month Randomized Control Design Study

**DOI:** 10.1371/annotation/e12c22d3-a42b-455d-9100-6c7ee45d58d0

**Published:** 2014-01-02

**Authors:** Pasquale Caponnetto, Davide Campagna, Fabio Cibella, Jaymin B. Morjaria, Massimo Caruso, Cristina Russo, Riccardo Polosa

The CONSORT checklist and trial protocol that accompany this study were erroneously omitted during the production process. Please view the files here:
