# Supplementary figures and images for: Correction: EffiCiency and Safety of an eLectronic cigAreTte (ECLAT) as Tobacco Cigarettes Substitute: A Prospective 12-Month Randomized Control Design Study
Source: PLoS One. 2014 Jan 2;9(1):10.1371/annotation/e12c22d3-a42b-455d-9100-6c7ee45d58d0. doi: 10.1371/annotation/e12c22d3-a42b-455d-9100-6c7ee45d58d0 (PMC3888441; doi:10.1371/annotation/e12c22d3-a42b-455d-9100-6c7ee45d58d0)

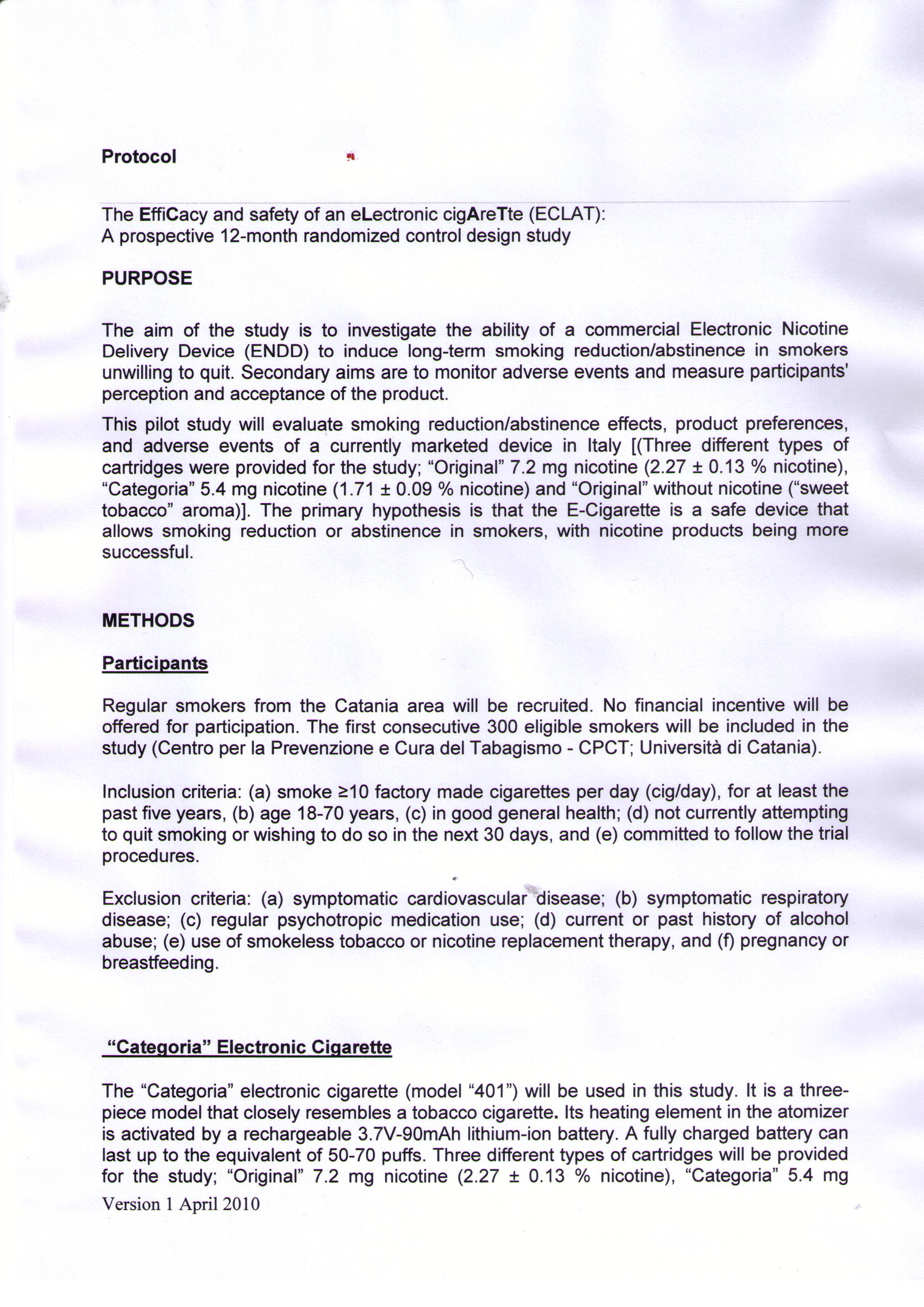

Supplement: Supplementary file 2 [file pone.e12c22d3-a42b-455d-9100-6c7ee45d58d0.s002.zip › protocol pag 1.JPG]

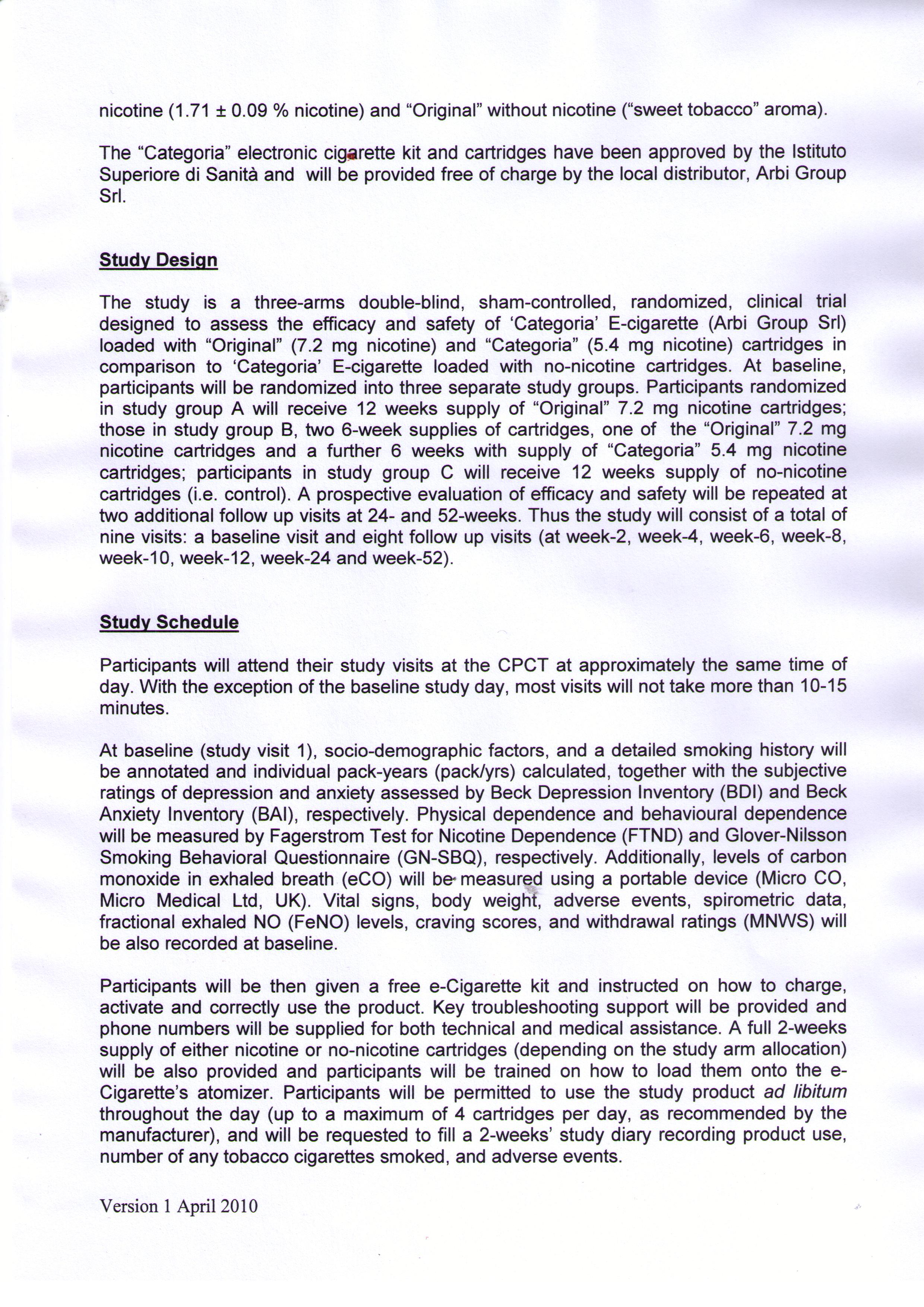

Supplement: Supplementary file 2 [file pone.e12c22d3-a42b-455d-9100-6c7ee45d58d0.s002.zip › protocol pag 2.JPG]

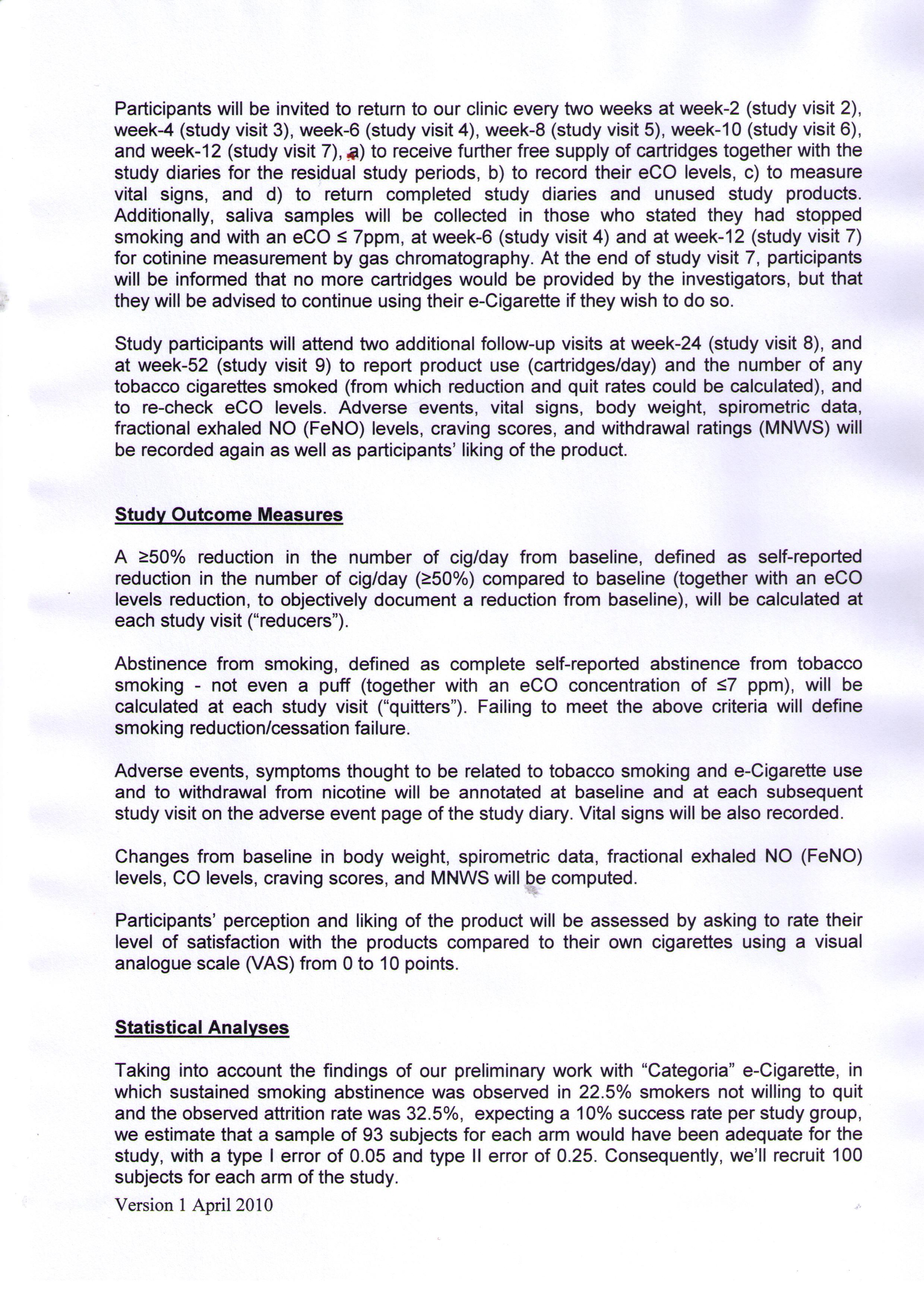

Supplement: Supplementary file 2 [file pone.e12c22d3-a42b-455d-9100-6c7ee45d58d0.s002.zip › protocol pag 3.JPG]

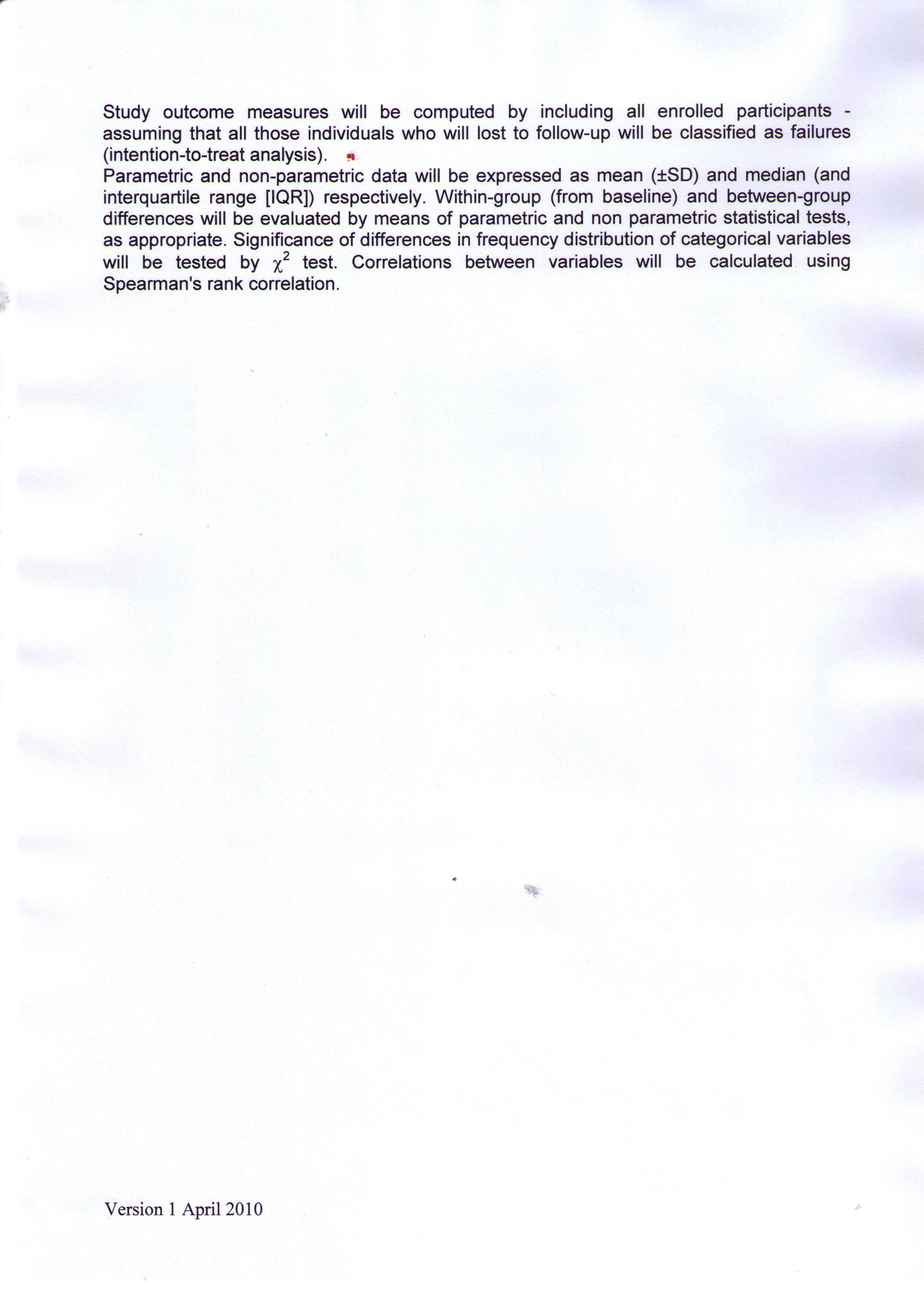

Supplement: Supplementary file 2 [file pone.e12c22d3-a42b-455d-9100-6c7ee45d58d0.s002.zip › protocol pag 4.JPG]

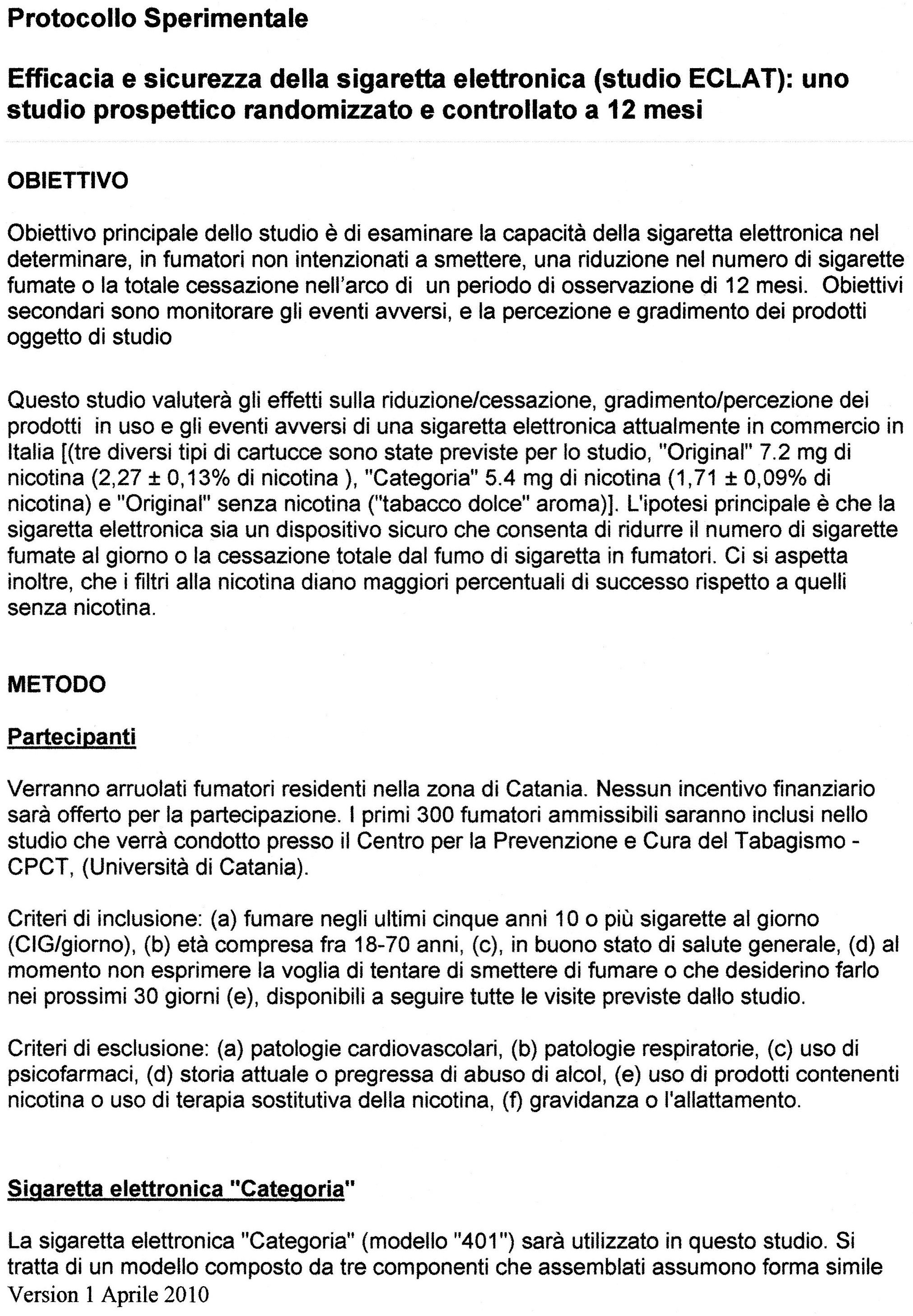

Supplement: Supplementary file 2 [file pone.e12c22d3-a42b-455d-9100-6c7ee45d58d0.s002.zip › protocollo sperimentale pag 1.JPG]

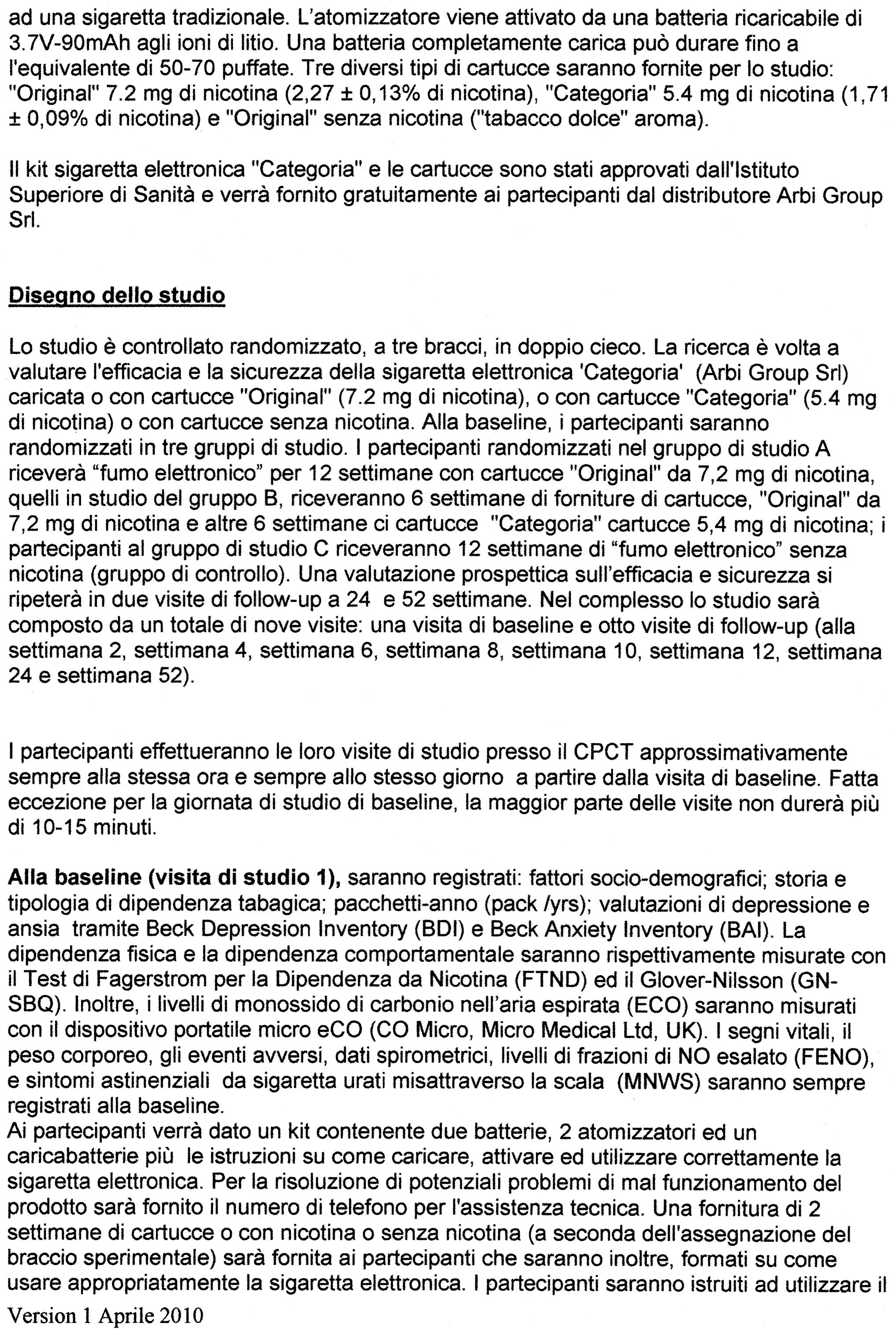

Supplement: Supplementary file 2 [file pone.e12c22d3-a42b-455d-9100-6c7ee45d58d0.s002.zip › protocollo sperimentale pag 2.JPG]

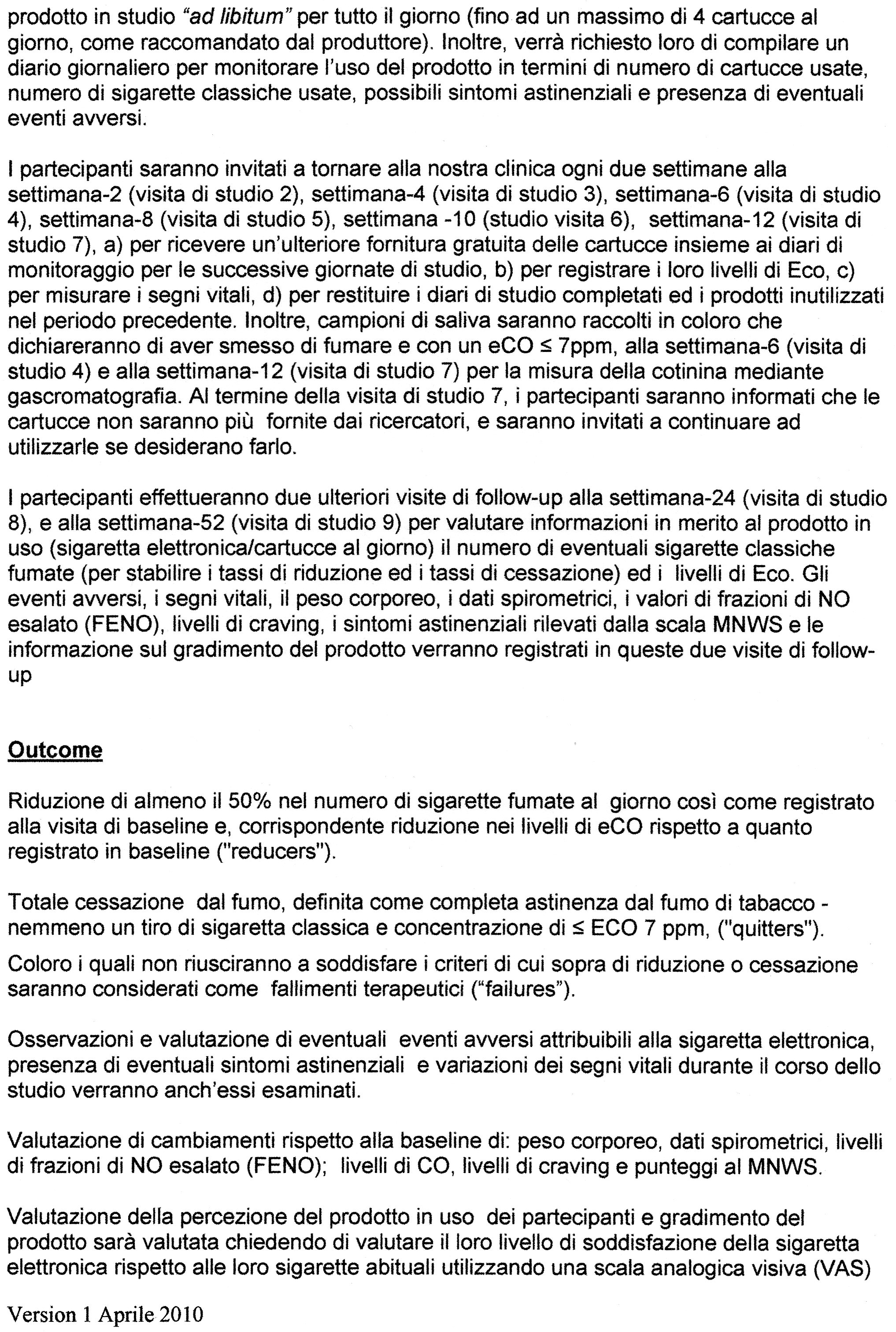

Supplement: Supplementary file 2 [file pone.e12c22d3-a42b-455d-9100-6c7ee45d58d0.s002.zip › protocollo sperimentale pag 3.JPG]

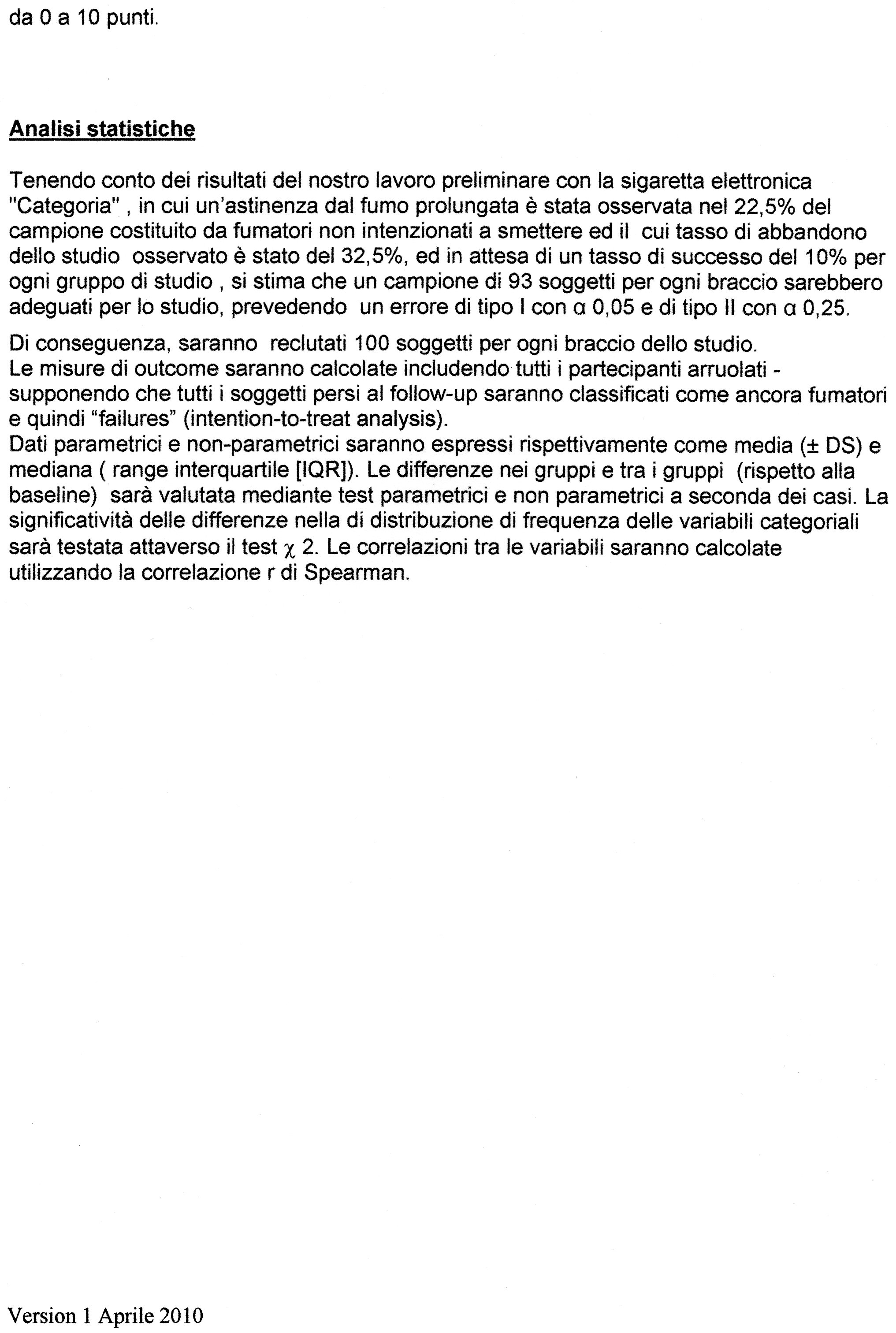

Supplement: Supplementary file 2 [file pone.e12c22d3-a42b-455d-9100-6c7ee45d58d0.s002.zip › protocollo sperimentale pag 4.JPG]
